# Supplementary material for: Trust in scientists and rates of noncompliance with a fisheries rule in the Brazilian Pantanal
Source: PLoS One. 2019 Mar 25;14(3):e0207973. doi: 10.1371/journal.pone.0207973 (PMC6433251; doi:10.1371/journal.pone.0207973)
Supplement: S1 Questionnaire — (DOCX) [file pone.0207973.s001.docx]

S1 Questionnaire

QUESTIONS / *PERGUNTAS*

Translations are not word-for-word, but were tested for understanding among community members in Brazil.

The fishery is in decline.

*Os peixes estão acabando.*

The decline of the fishery is caused by humans.

*Os seres humanos estão causando a redução de peixes aqui.*

It is a big deal when there is a violation of fishing rules.

*É grande coisa quando alguem viola as regras de pesca.*

The rule about pacu size is the correct limit to protect the fish here.

*A medida do pacú é a medida certa para proteger os peixes daqui.*

I trust the biologists to set the rules.

*Eu confio nos biólogos para definir as regras.*

The agency is successful in setting rules.

*O CEPESCA tem sucesso em definir as regras.*

The agency listens to me when creating rules.

*O CEPESCA me escuta quando estão definindo as regras.*

The agency respects me.

*O CEPESCA me respeita.*

The agency has similar values about saving the fishery as me.

*O CEPESCA tem valores semelhantes aos meus quanto à preservação da pesca.*

The agency treats everyone equally.

*O CEPESCA trata todo o mundo igual.*

The agency deceives me.

*O CEPESCA me decepciona.*

I will be caught by enforcement if I violate the rules.

*A policia vai me pegar se eu violar as regras.*

The penalty if I am caught is small.

*A multa se a policia me pegar é pequena.*

With what frequency will I break the rules in the coming year?

*Com que frequência irei violar a medida do pacú no ano que vem?*
